# Incentives for telehealthcare deployment that support integrated care: a comparative analysis across eight European countries

**DOI:** 10.5334/ijic.1062

**Published:** 2013-11-04

**Authors:** Maria Lluch

**Affiliations:** European Commission, Joint Research Centre (JRC), Institute for Prospective Technology Studies (IPTS), Edificio Expo, Inca Garcilaso, 3, Seville 41003, Spain

**Keywords:** integrated care, telehealthcare, incentives, European countries, information and communication technologies, organisation of care

## Abstract

**Introduction:**

Health care systems are struggling to deal with the increasing demands of an older population. In an attempt to find a solution to these demands, there has been a shift towards integrated care supported by information and communication technologies. However, little is understood about the role played by incentives and reimbursement schemes in the development of integrated care and information and communication technologies uptake. The objective of this paper is to investigate this question, specifically as regards telehealthcare.

**Methods:**

In order to identify the deployment of telehealthcare applications and their role in supporting integrated care, a case study approach was used. A clustering exercise was carried out and eight European countries were selected for in-depth study: Denmark, Estonia, Germany, France, Italy, the Netherlands, Spain and the UK. In total, 31 telehealthcare initiatives across eight countries involving over 20,000 patients were investigated.

**Results:**

Reflecting on specific examples in each initiative, drivers promoting integrated care delivery supported by telehealthcare mainstreaming and associated incentive mechanisms were identified. Attention was also paid to other factors which acted as barriers for widespread deployment.

**Discussion and conclusions:**

Trends towards telehealthcare mainstreaming were found in Denmark, the UK, and in some regions of Spain, Italy and France. Mainstreaming often went hand-in-hand with progress towards integrated care delivery and payment reforms.

A general trend was found towards outcomes-based payments and bundled payment schemes, which aimed to promote integrated care supported by telehealthcare deployment. Their effectiveness in achieving these goals remains to be seen.

In addition, a form of outpatient diagnostic-related group reimbursement for telehealthcare services was found to have emerged in a few countries. However, it is questionable how this incentive could promote integrated care delivery on its own.

This research suggests that incentives which align social, primary and hospital care are rare and there is a need to design new payment paradigms.

Finally, eHealth penetration, interoperability, governance, availability of evidence and reorganisation of services represent additional factors which can act as drivers or barriers for integrated care delivery.

## Introduction

The importance of chronic disease prevention and management becomes clear when the combined effects of the projected ageing of the Organisation for Economic Co-operation and Development population, the limited capacity of existing health care systems to deal with the increasing demands of an older population and the continued rise in health care spending are considered [1,2]. In response, several countries have been experimenting with new models of care delivery in an effort to achieve better coordination of services across the continuum of care. These models range from disease management programmes developed in the late 1990s, to the most recent trends towards integrated care [3,4].

Indeed, the pressure on health systems, which creates the need for care in the home and in the community whenever possible, could ultimately promote coordination or even integration of health and social services [5–8]. This shift in the balance of care towards the home environment offers enhanced primary and community care, which focuses on helping patients to care for themselves, rather than on secondary care [9].

eHealth can support the development of integrated models of care because it can integrate multi-professional teams and institutions through information sharing [10] thus enhancing service coordination. Moreover, eHealth and telehealthcare in particular can help patients to manage and monitor their own diseases to a greater extent [10,11].

Telehealthcare is defined as personalised care delivered by both synchronous and asynchronous (such as store and forward) technologies over distance, which enables data to be transferred from the patient to the professional, who then provides feedback [12].

Successful experiences were initially found in the US Department of Veterans Affairs Care Coordination Home Telehealth programme. Through patient-centred care coordination and supportive telehealthcare technologies, patients were routinely monitored and educated about their diseases by care coordinators. In addition, care coordinators also acted as brokers with the care system if intervention was required. The Veterans Affairs programme proved to be successful in preventing more costly interventions [13,14].

Another example of telehealthcare deployment was provided by the Whole Systems Demonstrators. A patient-centred system redesign, which promoted self-management and coordination across tiers of care, was undertaken [9]. Evaluation after a 12-month trial found relevant benefits for telehealthcare deployment [15,16]. These will be detailed later in this paper.

Despite the difficulties identified in mainstreaming telehealthcare applications as a constituent element of integrated care [11], recent trends point towards a proliferation of telehealthcare-based interventions, underpinned by evidence of variable quality [12]. However, their sustainability still faces a series of challenges: for example, the level of information and communication technologies uptake, interoperability, liability issues, accompanying policies promoting cooperation across tiers of care and aligned incentives.

The role played by incentives to service providers and/or purchasers is particularly challenging. Incentives can ensure continuity of care between the hospital, primary and social care systems. They are seen as central to the facilitation of mainstream implementation of telehealthcare and more generally integrated care [3,7,11,17–20].

The objective of this paper is to investigate the role played by incentives and reimbursement schemes in the development of telehealthcare applications to support the delivery of integrated care. To this end, experiences in eight European Member States were studied in depth, namely Denmark, Estonia, Germany, France, Italy, the Netherlands, Spain and the UK. The findings reported here have been taken from a broader research project (SIMPHS2 [21]).

For the purposes of this paper, the term ‘incentive(s)’ will be used in its wider sense, to include any aspect of an organisation that may lead to particular behaviour in relation to telehealthcare deployment, cooperation between tiers of care and the delivery of integrated care. Furthermore, the term ‘tiers of care’ will refer to tiers across the health care domain (primary, specialised and hospital care) and social care, given that integrated care concerns both the health and social care sectors [22–25].

## Methods

In order to identify deployment of telehealthcare applications and their role in supporting integrated care, a case study approach was used [26–28]. Following a clustering exercise, the eight European countries selected for in-depth study were Denmark, Estonia, Germany, France, Italy, the Netherlands, Spain and the UK. The selection was based on these countries’ health care organisations and eHealth readiness and, at the same time, aimed to reflect the diversity of European Member States. To enrich the results, a regional approach was followed in each country in line with previous and current developments in the field [2,11,29].

### Data collection

The methods involved a combination of secondary and primary data collection.

Secondary data collection was carried out through desk research to gather: socio-demographic statistics (e.g. total population, recent demographic trends and forecasts, age structure, socio-economics and education); prevalence statistics with special focus on chronic conditions; information on health care organisation (e.g. policy context, health care spending, financing, incentives and disease management programmes); information on social care organisation; evidence of cooperation across different tiers of care; and data on the eHealth context (e.g. investment levels, adopted applications, penetration rates, deployment levels and data exchange). Particular emphasis was given to developments with shared Electronic Health Records as the central component of an integrated health information system.

Criteria for selecting case studies included evidence of cooperation between tiers of care, a relatively advanced level of implementation in terms of sustainability and population reach, and also the presence of a telehealthcare component. In addition, the uniqueness of some of the experiences and research access were also used as criteria for inclusion so as to obtain relevant data in each country targeted.

Primary data collection in each country consisted of the identification and selection of a minimum of two specific relevant case studies. Subsequently, direct observation was performed. In addition, a minimum of 10 key stakeholders at regional or national level were interviewed: policy-makers and government officers; representatives of Health Technology Assessment agencies; managers of health care centres or project managers of specific initiatives; health and social care practitioners; technology providers; and patients.

Through semi-structured interviews with stakeholders, qualitative data were obtained on the following topics: How the experience was initiated? What factors triggered development? Who was involved and how it was operationalised? How the innovation was diffused and/or disseminated? plans for mainstreaming; drivers and barriers encountered and the challenges ahead. Transcripts for each interview were made and, where appropriate, translated into English.

In total, 31 telehealthcare initiatives across eight member states involving over 20,000 patients were the object of this analysis. The data collection resulted in the production of eight country's studies in 2012 which were published as individual reports [30–37].

### Data analysis

Following completion of the reports, a synopsis for each country was developed covering all aspects related to incentives which could influence telehealthcare deployment and progress towards integrated care. In particular, the following aspects were selected from each of the country reports: organisation and purchase of care services; payment schemes to care organisations; financial rewards to care professionals related to performance, activity, information and communication technologies and cooperation. Where appropriate, the findings were completed by a literature review. This was particularly necessary for the Netherlands. In addition, recent trends in health care reform that could further influence progress towards integrated care and incentives were also included.

Content analysis from the interview transcripts and subsequent coding allowed the selection of aspects related to incentives.

Finally, a summary was developed for each experience in an inductive process, using the results from the interview analysis and the synopses for each country, descriptive details of each implementation and scaling-up, trends and policy issues related to incentives. A selection of these are the objectives of the following sections, which aim to emphasise the gaps and good practices in incentives and reimbursement mechanisms for telehealthcare services and integrated care.

## Results

The clinical conditions most commonly targeted across experiences were chronic obstructive pulmonary disease, heart conditions and diabetes. Frailty was also one of the targets. Details of telehealthcare deployment in each of the experiences in all the countries studied are covered in Table 1.

The tier of care initiating the experience varied from primary care, to hospital care or social care. Leadership across initiatives often reflected specific features of health and social care system organisation. For instance, Germany and the Netherlands provided a couple of cases where insurance companies were involved in launching the initiatives. These cases reflect the role that insurance companies or sickness funds play in these countries.

As regards telehealthcare mainstreaming, trends were found in the UK, Denmark, France, and in some Italian and Spanish regions also. Of paramount importance is the fact that cooperation cross tiers of care were also identified in Denmark, Italy, Spain, the UK and to some extent Germany.

Using illustrative examples of the experiences researched (see full list in Table 1), the rest of this section outlines the different incentive mechanisms across tiers of care in each of the countries (see Table 2).

### Denmark

In Denmark, two experiences targeting chronic obstructive pulmonary disease patients were found, namely Telekat and the Patient Briefcase [35]. The significant socio-demographic costs of chronic obstructive pulmonary disease, mainly accrued by hospital admissions (and readmissions) and ambulatory visits and, to a lesser extent, the costs of visiting the general practitioner, represented a driver for these initiatives. These two hospital-level initiatives aimed to prevent the readmission of chronic obstructive pulmonary disease patients by offering them tele-homecare (telehealthcare) technology to help them with rehabilitation in their own homes.

In both cases, when a patient was discharged from hospital and given telehealthcare technology, home nurses from the municipality were informed. Thereafter, collaboration between all the health care professionals involved took place in accordance with a specific cooperation agreement made for the course of treatment. During the initial weeks at home, telehealthcare data were monitored by nurses and doctors from the corresponding hospital unit. They guided the patients in dealing with their condition.

After this period, the patient was transferred to regular home nursing and the home nurse in the municipality received a standardised status report on inclusion of the patient in community care. The patient's general practitioner was also informed about the patient's transfer and health status. Approximately four weeks after discharge, the telehealthcare equipment was removed and the patient was offered a minimum of one phone follow-up and the standard ambulatory follow-up received by all chronic obstructive pulmonary disease patients at the hospital.

Both experiences proved so successful that the hospital leading the Patient Briefcase initiative now aims to expand the service into other conditions such as diabetes or early discharge of mothers with new-born babies. Meanwhile, the Telekat initiative is being mainstreamed throughout the North Denmark region.

Another initiative is the recently launched Integrated Clinical Home Monitoring Project. This is a telehealthcare project that will test the use of home monitoring on various chronic illnesses, share data and communicate across traditional sector boundaries, while developing common electronic communication standards. It is a cross-sectorial, technical, and organisational project which focuses on integrated care above all. Integrated Clinical Home Monitoring Project involves a number of municipalities and general practices across two of the five Danish regions.

From an incentive and coordination perspective, the hospitals falling under the responsibility of these two regions benefit by avoiding readmissions which are often associated with long stays. In addition, since 2011, hospitals receive reimbursement from telehealthcare activities through DAGS, a form of outpatient diagnostic-related group. In contrast, no financial incentives were identified in the initiatives studied at primary care level. However, since general practitioners in primary care are contracted by the regions, it is in their interests to be cooperative.

Home nurses and their municipalities only benefit economically from telehealthcare when distances to patients are long. Nevertheless, health care agreements between regions and municipalities may act as an incentive as these aim to ensure coordination, coherent patient pathways and rehabilitation support at community level.

### Estonia

Virtu (virtual elderly care services) was launched in some Estonian municipalities on the Baltic islands. It aimed to improve the quality of life for elderly and handicapped people in these areas [30].

The objective of Virtu was to help the elderly in the archipelago area to live at home, support their social interaction, improve their quality of life and increase their safety. This was achieved through teletraining and teleconnection between homes (participant to participant) and between homes and carers (or doctors). From the provider side, the main users were social workers and homecare givers. It was expected that primary care, such as family (general) physicians, would also be involved at some point.

Prior to Virtu, social workers used to monitor the elderly by phone calls and home visits. The expectation was that implementing the Virtu technology would save money on treatment and care. Using virtual conference technology enabled social workers to check up on elderly people's health, well-being and living environments without actually travelling to their homes. However, it was very difficult to see how municipalities could pay for this service after the pilot project. The financing of home care services in Estonia has not changed for almost 15 years and municipalities currently have problems covering even the most basic needs of their people.

This problem is further aggravated by the smallness of Estonian municipalities which make economies of scale for any service beyond basic first level (e.g. telecare alarm buttons) difficult to achieve.

Contrary to expectations, general practitioners did not become involved even though Virtu's design provided a role for them at some point. Incentives for telehealthcare at primary care level are weak, given that family doctors and nurses, who act as gatekeepers in primary care, are contracted by the Estonian Health Insurance Fund via a combination of capitation payments (76% of their income) and other remuneration, including fee-for-service and capital costs. More recently, a performance payment was introduced which aimed to improve the quality of care.

There are also few incentives for hospitals to focus on replacing hospital bed-days with telehealthcare applications because the hospital revenue model is based on Estonian Health Insurance funding combining payments based on the diagnostic-related group and the days spent in hospital. This is probably one of the main reasons why hospitals have little interest in new information and communication technologies-enabled monitoring services, although there have been a couple of positive cases in the past. In these cases, the innovative image acquired through participation in a telehealthcare initiative was a major driver for the hospitals concerned (namely East-Tallinn Central Hospital and Tartu University Hospital).

### France

The Esoppe programme is one of the regional geriatrics experiences of the Limousin region in France [37]. Esoppe is part of a territorial strategy to help the elderly live safely and comfortably at home. It provides a home automation system which helps prevent elderly people from falling, especially when they get up during night, through automatic lighting devices adapted to home layout. Esoppe is based on ambient-assisted living technologies which control and monitor possible falls through fall sensors. Dissemination of this service was greatly facilitated by regional authorities taking an active role as stakeholders and by the involvement of the Ministry of Economics, Finance and Industry. This gave further signals to the market about the need to search for new economic models outside of the traditional compulsory health care insurance schemes.

Other cases in France where public authorities or medical networks have been involved have been widely deployed and the innovation has been diffused rapidly, highlighting the importance of the public–private partnership in succeeding with telecare. These cases also show that it is possible to establish business models without relying solely on reimbursement. Up until now, this feature has not been identified in telehealth experiments and the issue of reimbursement is a recurring barrier mentioned by both payers and industry players.

Indeed, it seems that successful telehealthcare experiences in France have had to operate almost outside the health and social care systems, as current incentives in these systems do little to promote telehealthcare uptake.

Hospital reimbursement is based on diagnostic-related group-like tariffs for inpatient care whilst ambulatory services are reimbursed through fee-for-service schemes. Recent reforms, however, have been introduced which promote care at home reimbursed on a diagnostic-related group basis for outpatient care. In addition, reimbursing telehealthcare services on the same basis has been put on the agenda.

At primary care level, physicians were traditionally paid per number of consultations. In 2011, however, an agreement was reached on a new incentive system which focuses on quality of care through outcome-based payments, rather than number of consultations. The indicators used as the basis for these incentives take into account the implementation of the disease management programmes, ePrescription use, and information technology adoption to enable transmission and use of teleservices.

Finally, long-term care for the elderly and disabled belongs to a specific sector within the social system referred to as the ‘health and social care sector’ or ‘third sector’. Here, community nurses are entirely financed by Social Health Insurance on a case-payment method, adjusted to the patient's level of need.

### Germany

Telehealthcare development in Germany appeared to be limited. Pilots exploring its potential were usually carried out by sickness funds. An exception was the WohnSelbst initiative, which was led by a hospital and a social housing association in the city of Wiesbaden [31].

WohnSelbst explored the possibility of patients’ homes becoming the primary location of health care delivery, by extending regular monitoring for health care purposes to residential housing.

Serious marketing efforts including letters, events, face-to-face contact and vouchers were made in order to recruit users. Only 35% of those targeted took up the service even though it was free of charge. Initially, it was assumed that general practitioners would become involved in the project and would help with patient recruitment, but, in the end, they did not.

Scepticism from general practitioners and specialists at primary care level was driven by a variety of issues, for example, the lack of a tradition of cooperation with other tiers of care. They were also suspicious of hospitals becoming involved in tasks which traditionally were performed by primary care. In addition, financial aspects played a part, given that general practitioners offer health checks to patients as a non-insurance service (individual health service) paid for privately (around €37). WohnSelbst was offering an initial health check to each patient who enrolled in order to assess their needs, which was seen as an intrusion by primary care doctors and specialists.

As initial recruitment was so poor, the initiative was extended to the city of Taunusstein and surrounding villages in the Rheingau-Taunus municipality where greater uptake was expected. Interestingly, the mayor of Taunusstein became a champion of the project and, at an information event for patients, 30 new residents signed up.

The future for WohnSelbst is, to some extent, uncertain once the funding received from the Ministry of Education and Research is exhausted. There is, however, potential to develop a business case for its continuation as a commercial service.

The key lesson from the WohnSelbst project is that initiatives of this kind will struggle unless patients’ general practitioners and/or specialists are involved. This can be achieved through creating awareness and involving them as early as possible in order to get them to buy-in. However, unless there are also financial incentives in the form of reimbursements for services provided, clinicians are unlikely to become involved.

Moreover, separation across tiers of care is still strong, both in terms of organisational processes and embedded cultural attitudes. Organisations share information on patients via letters, but there are no further links and the sectors have little incentive to manage patients jointly.

Competition between individual organisations and the tiers of care and a lack of shared budgets are further disincentives to providing integrated care.

Indeed, health care in Germany is funded by a statutory contribution system which covers around 90% of the population via the 43 operating health insurance funds. Outpatient services (access to general practitioners and to specialists) are still largely the responsibility of independent clinicians practising on a freelance basis under contract with the statutory health insurer. These services are frequently reimbursed as a mix of capitation and fee-for-service, often adjusted by patient condition. In 2002, structured disease management programmes were introduced in Germany. However, these programmes offer general practitioners only moderate incentives. At hospital level, reimbursement is a mix of fee-for-service and lump-sum payments calculated according to diagnostic-related groups.

Integrated care gained importance when the contract law between insurance providers, doctors and hospitals became more flexible. This law required every insurance provider to retain up to 1% of total statutory contribution system funding from physicians and some hospital invoices, as long as this budget was needed for the implementation of any integrated care contracts.

Finally, social care is the responsibility of the *Länder* (one of the 16 sovereign constituent states of the Federal Republic of Germany) and is delivered by a wide variety of mainly private organisations that complement family and lay support.

Thus, efforts to promote coordination and continuity across tiers of care exist, but this research suggests that they are limited.

### Italy

In Italy, the eCare network represents one of the most successful initiatives identified in the study [32]. The service began in 2005, and is currently being delivered to over 11,000 elderly frail people, who are watched over by a specialised service centre delivering tele-monitoring, tele-assistance and tele-company services. This centre remains in constant touch with both social and health care services delivered by the Local Health Authority. Through routine monitoring, the aim is to prevent the aggravation of social and health frailty, and avoid unnecessary hospitalisation by early detection of a decline in the condition of the elderly people concerned.

The eCare network involves a range of services including a 24-7 call centre; a socio-sanitary dossier recording contacts and updates for each user; at least one phone call a week according to the Individual Assistance Plan; electronic bookings of medical visits and exams; links with general practitioners and clinicians; access to and organisation of social services (transport, accompaniments, shopping); calls to emergency services (policemen, firemen, …) and agreements with craftsmen to provide home maintenance works.

Enrolment can be done by any of the authorities that participate in the project: the AUSL Bologna (the local health enterprise responsible for ensuring the delivery of health care), local general practitioners (so far, however, they have not been really active contributors) and nurses; social service workers and councils/ municipalities; and non-profit organisations.

Given the high number of citizens and organisations involved, a technical committee was established supported by specialty boards responsible for redefining the services for the different types of users enrolled. For instance, a specific board was recently set up to refine protocols and selection criteria for users with cardiovascular diseases.

Since 2007, eCare has been financed through the fund for non-self-sufficient people (fondo regionale per la non autosufficienza). It is therefore included in the Bologna district's social services financial resources, the district's municipalities and AUSL Bologna.

The integration of different domains such as health and social services is the real added value of the service. Each user report is truly multidisciplinary and it involves social workers, nurses and to some extent physicians. eCare is a ‘community health service’ in the truest sense of the word. Both clinical and social indicators are taken into consideration. As regards the social aspects, it is important to highlight that the service benefits from a significant contribution from volunteers of non-profit organisations.

Although funding was available to mainstream this initiative and the commitment from the local health management organisation (ASL) assisted the implementation and user recruitment, general practitioner involvement has been passive. Italian general practitioners and paediatricians, acting as gatekeepers, deliver primary care and preventive medicine. They are paid in a mix of capitation and a variable payment which aims to reward them for effective cost containment. These costs could comprise fees for services for specific treatments, including minor surgery, preventive activities, therapies, post-surgery follow-up and the delivery of planned care to specific patients, such as home care for chronically ill and disabled people.

At hospital level, payments for inpatient and outpatient care are established by means of predetermined rates based on diagnostic-related groups, whilst hospital physicians earn a monthly salary. Little evidence of hospital involvement has been identified in this initiative.

The role of the municipalities, traditionally responsible for organising the delivery of social care, and voluntary organisations has been key to the success of this project.

Other interesting telehealthcare experiences in Italy, such as my doctor@home and NRS (formerly telemaco), were identified. These began in hospitals and mainstreaming towards primary care was facilitated by the development of diagnostic-related group payments for outpatient care (i.e. CReG in Lombardy, Italy) which promoted telehealthcare deployment.

### The Netherlands

Three cases were selected from the Netherlands, namely TEHAF study - Health Buddy; COPDdotCOM and Cardioconsult [36]. The Koala project was also covered to some extent. An aim common to all these cases was to explore the role of information and communication technologies in disease management and to conduct cost-effectiveness studies. Hospitals played the main role in all these cases with little involvement from other tiers of care.

It is unlikely that any of the services concerned will be mainstreamed. However, some trends towards integrated care were identified. The government pledged €340 million for pilot projects in chronic care (not all information and communication technologies related) for the period 2005–2013. In addition, ZonMW (the Netherlands organisation for health research and development) is running activities in information and communication technologies and disease management (COPDdotCOM was one of the projects included). Also, ZonMW launched an integrated care programme for diabetes which is experimenting with a new payment system for integrated diabetes care. The new system is used as an instrument to stimulate the formation of integrated multidisciplinary groups of professionals in diabetic care. Finally, the recently introduced ‘bundled payment’ [38] may pave the way to integrated care developments and telehealthcare deployment.

Indeed, the Dutch health care system is organised around multiple competing private health insurers and until recently, payment negotiations with health care providers were based on price, volume and quality of care.

The introduction in 2010 of ‘bundled payment’ which promotes the coordination of care for chronic conditions represents a significant milestone as regards integrated care [39]. In this bundled payment model, health insurers pay a single fee to a principal contracting entity. This new legal entity called a care group covers all elements of primary care for patients with a specific chronic disease and serves as the general contractor which itself delivers the various components of care or subcontracts with other health care providers (practitioners, laboratories, dieticians and specialists) to deliver them. Care groups consist of multiple health care providers and are often owned by general practitioners. This bundled payment, being voluntary, aims to overcome the barriers to integrated care which resulted from the traditional payment system to general practitioners (combining capitation and fee-for-service payments).

However, the role of social care services under this payment mechanism is not clear.

### Spain

Two initiatives in Spain (one in the Basque country and one in Catalonia), which transfer telehealthcare from hospitals to primary care, were identified [33].

Experiences in Andalusia were also explored, given the high deployment of ehealth applications (ePrescription, Electronic Health Records, etc.) across all primary care and ambulance services. However, telehealthcare experiences were very limited.

In the Basque country, the experience in the evidence-based Medicine Clinical Unit of the Donostia Hospital (San Sebastian) was transferred to primary care through the project Telbil. It is important to mention that health care professionals involved in Telbil were very satisfied with the ‘new communication channels between primary and secondary care’. However, Telbil was a small-scale pilot that relied on a clinical champion at primary care level. After evaluating the results, evidence was provided on the effectiveness of the intervention. It was concluded that in order to mainstream telehealthcare at primary care level, a reorganisation of health and social care and the incentives systems was required. These changes are at the core of the current health strategy in the Basque country. They have evolved from activity-based to outcome-based payment for securing better health outcomes. They have also gone from a hospital-centred model to a patient-centred model and from a fragmented model to a model which enables continuity of care through the integration of health and social care services.

In Catalonia, the Nexes project built on earlier proof-of-concept pilots at Hospital Clinic. It was a deployment project (with almost 3,000 patients) which aimed to transfer patients from hospital to primary care and their homes with information and communication technologies support.

Nexes acknowledged that fragmentation between health and community services represents a major problem.

The four integrated care services considered in Nexes were: wellness and training, enhanced care for frail patients, home hospitalisation and early discharge, and remote support to primary care for diagnosis and therapy. Thus, many of the activities promoted self-management and prevention activities whilst coordination across tiers of care enhanced the care continuum.

Nexes identified positive health outcomes and enhanced use of health care resources, which made outpatient care a better strategy than the use of inpatient services. The need to define a reimbursement system that represents the interest of the payer was acknowledged as a result. The final scenario could be a reduction in inpatient services costs (estimated at 17% in this experience) and these savings could be spent in the form of home care and information and communication technologies innovation. The overall spending could either be reduced or health care outcomes increased with the same expenditure [40].

Indeed, financing for hospital care in Spain uses prospective reimbursement mechanisms such as diagnostic-related group tariffs in combination with other payments such as capital payments. In contrast, general practitioners act as gatekeepers and their pay consists of partly a fixed salary and partly a combination of capitation and payments for on-call shifts performed, seniority and most recently, for achieving performance quality indicators which include adherence to chronic disease management guidelines. Therefore, as acknowledged in Nexes, a reform of National Health Service financing and reimbursement mechanisms would assist in integrated care service delivery.

In Andalusia, an interesting outcomes-based payment system along the same line has recently been introduced. It aligns incentives for health care professionals at district level across primary and hospital care.

Instead, social care services rely upon regions, municipalities or national reference centres strongly supported by Non Government Organisation networks. Currently, coordination between health and social care mainly takes place through primary care although recent reforms aim to strengthen coordination between these two tiers of care by establishing targets and indicators.

### The UK

The study in the UK focused on England and Scotland, each of which are responsible for their own health and social care. The term UK will be used when referring to common findings in these two countries. Where a feature is specific to only England and not Scotland or vice versa, this will be duly clarified.

The interest in telehealthcare solutions was driven by the anticipated challenges of providing care for the UK's increasingly aging population: 17.5 million adults have chronic illnesses - a figure set to rise to 35 million by 2030 [34].

Highly influenced by the positive experience of the US Veterans Health Administration, the case for telehealthcare solutions grew, and a number of successful pilots and trials took place across both England and Scotland.

Both countries moved from numerous pilots, often primary-care driven, to widespread deployment.

In Scotland, deployment of telehealthcare was closely related to the adoption of the National Telecare Development Programme which not only aimed to promote the use of telehealthcare technologies but also to coordinate health and social services at primary care level. Indeed, one requirement for obtaining Telecare Development Programme funding was that health and social care services had to submit a joint application as a local partnership. Thus, the Telecare Development Programme aimed to get these two tiers of care to work together and provide integrated services to patients.

In each Telecare Development Programme pilot, telehealthcare implementation and day-to-day activity were mainly driven by trained, community nurses. For the pilots, community nurses received additional, specific training about the conditions they were dealing with and about the telehealthcare technology. They were also responsible for training the patients. Patient-level data collected through telehealthcare readings were not integrated with their Electronic Health Records. However, in many of the experiences community nurses also had access to Electronic Health Records, often using a remote desktop application. This allowed nurses to better assess the condition of each patient and better coordinate each case. In addition, community nurses would also coordinate with social care services, thus achieving the desired coordination between primary care and community nursing [41].

In England, telehealthcare was deployed on a large scale through the Whole Systems Demonstrators. Two-year research projects at three sites (Cornwall, Kent and Newham - chosen for the diversity of their populations) were funded by the Department of Health. Over 6,000 participants were allocated at random to control or intervention groups and followed up over a 12-month period. The aim of the Whole Systems Demonstrators was to explore the role of telehealthcare in reducing the use of health services by people with long-term conditions and social care needs [9].

Results from the Whole Systems Demonstrators concluded that telehealth is associated with lower mortality and emergency admission rates [15].

Based on these experiences, both countries concluded that joint benefits in terms of costs, effectiveness and helping patients to stay in the community were reaped from health and social care working together supported by telehealthcare. With funding through the Dallas programme (delivering assisted living lifestyles at scale), mainstreaming is currently underway.

Traditionally, the coordination between health and social care was mainly supported at local level through community nurses employed by English Primary Care Trusts or Scottish Health Boards working together with local authority partners (e.g. social services, housing, leisure). More recently, competences for adult social care have been shifted to the pertinent Department of Health, in order to integrate services for adults and support telehealthcare mainstreaming.

Furthermore, independent primary care practices contracting with the National Health Service, who represent the primary care base and act as gatekeepers, are paid through a combination of capitation schemes and outcome-based incentives defined by the Quality Outcome Framework. The Quality Outcome Framework defines and binds primary care practices’ financial income to a set of criteria and population outcomes delivered by the practices. These incentives may actually encourage the deployment of telehealthcare applications.

However, active involvement of general practitioners in these experiences was very limited and most of the telehealthcare activity was left to community nurses. Nonetheless, current trends may influence mainstreaming plans.

Trends in Scotland indicate a strengthening of the role of community partnerships. Funding has been made available for these partnerships, which provide health and social care and represent the care unit at local level. England is opting instead for the establishment of clinical commissioning groups, which are often established and led by primary care practices and are likely to hold the contracting role of Primary Care Trusts at local level.

From a hospital perspective, payment-by-results schemes were introduced as a form of diagnostic-related group tariff to reimburse hospital services. Payment-by-results tariffs apply to elective care, emergency care and outpatients. Due to recent reforms implemented in England, a hospital readmission within 30 days of discharge is not eligible for hospital reimbursement.

## Discussion

Most experiences show that telehealthcare deployment is initially influenced by the availability of funding, not only as a driver to test or scale it up but also as an incentive to promote integrated care and the sustainability of the initiative concerned.

However, telehealthcare developments should not be looked at in isolation or as silos but in the context of other incentives in the health and social care system. This could shed some light on ways of progressing towards the delivery of integrated care and on how telehealthcare services could support care.

In Denmark, the Integrated Clinical Home Monitoring Project represents deployment on a large scale involving home monitoring for 2,000 patients with chronic obstructive pulmonary disease, diabetes or inflammatory bowel disease and also pregnant women. As seen, the incentives at hospital level are driven by the long hospital stays for patients with certain conditions which represent high costs, not necessarily offset by diagnostic-related group payments. In addition, the hospitals’ drive to innovate towards new forms of care also acts as a facilitator. Furthermore, DAGS reimbursement for telehealthcare as an outpatient care activity represents an additional incentive.

In contrast, incentives at primary and community care levels are limited. Nonetheless, the fact that general practitioners contract with regional authorities, which also manage hospitals, may positively influence cooperation. In addition, the cooperation agreements between regional health care authorities and municipalities responsible for social care act as another incentive.

Direct incentives for telehealthcare at primary and community care level only seem appropriate when long distances exist between patients and the community, given the associated time savings in these instances. Indeed, this limitation is reflected in some of the Danish initiatives, where telehealthcare equipment was removed from patients after four weeks following hospital discharge and replaced by weekly phone calls. It could be argued that telehealthcare monitoring for longer periods, instead of only for a short time after hospital discharge, could lead to better patient outcomes. Although additional evidence would be required to prove such a hypothesis, DAGS can be seen as an incentive to promote telehealthcare adoption and cooperation with tiers of care in the short run. However, DAGS may not necessarily promote long-term cooperation between primary and hospital care and other types of incentives may be required.

In Estonia, high project mortality was observed. Telehealthcare only seemed an appealing option from a social care perspective where distances were long. In addition, there was a clear lack of both funding and incentives.

These issues can be largely addressed by the state in a top-down approach, which would benefit from the potential savings in replacing hospitalisation with other types of care (e.g. telehealthcare-supported home care). Nevertheless, the state has been slow to take further steps in instituting telehealthcare services and the status quo of traditional hospital-centred health delivery has so far proved difficult to shake. Therefore, it appears that though innovation of services is considered a driver on paper and in strategies, the facts point in a different direction. Policies promoting integrated care are currently lacking and there is a palpable need for central government institutions to play a role in developing them. Integrating nationwide information and communication technologies health and social systems would be a step forward, and at the same time, incentives across tiers of care should be redesigned to promote cooperation.

In France, the efforts of regional authorities and the Ministry of Economics, Finance and Industry, have resulted in programmes with deployment on a large scale. Nonetheless, these rarely target health monitoring directly and focus more on highly advanced telecare applications.

Furthermore, telehealthcare deployment operates in parallel to health and social care systems rather than being embedded within them, though this may change in the nearer future in the light of recent reforms. First, outcomes-based incentives were implemented in primary care. Second, the role of the ‘third-sector’, which has existed since the 1970s, has recently been strengthened through the Regional Health Agencies. Third, funding has been made available to use and mainstream telehealthcare services with industry involvement. Finally, a diagnostic-related group-tariff for telehealthcare services as an outpatient service has been established. Therefore, it could be argued that once these recent developments are consolidated, a convergence between telehealthcare deployment and health and social care systems may take place. Synergies across both these systems may enhance further deployment, which would support integrated care.

Experiences in Germany provide examples of only moderate incentives to promote disease management programmes, and even less cooperation across tiers of care. Although new contract laws represent an effort towards integrated care, insurers have not yet developed appropriate incentives to promote either cooperation across tiers of care or telehealthcare deployment.

In Italy, there are incentives to facilitate telehealthcare deployment supported by the fund for non-self-sufficient people and in some regions by a form of diagnostic-related group reimbursement for outpatient care. Nonetheless, despite the success of experiences like eCare, general practitioners are only moderately or passively involved. In addition, there is little evidence of the involvement of social care services in other experiences which were initiated at hospital level and then transferred to primary care.

In the Netherlands, it is likely that the recent introduction of the bundled payment has generated a situation of uncertainty which hampers the adoption of new innovations (i.e. telehealthcare) and new developments towards integrated care. This may contribute to the high project mortality observed. However, now that this new payment mechanism is being consolidated and reshaped as a result of recent evaluations, greater confidence among care groups may result in progress towards integrated care and telehealthcare adoption.

In Spain, the two cases suggested that action needs to be taken as regards incentives which promote integrated care and telehealthcare deployment. The Basque country introduced activity-based payments and an integration of health and social care services.

Nexes in Catalonia also raised the need for new reimbursement models. Specifically, Nexes proposed the use of funding and/or new business models promoting innovation in integrated care supported by information and communication technologies.

Across the UK, the Dallas project (launched in 2012) has been deploying independent living products and services on a large scale. Dallas tasked four consortia with delivering this programme across communities to positively impact on the lives of 169,000 people by June 2015 by providing an extended range of services, focusing on population health, tailored care for multi-morbidity, clinical governance, professional management and staff career development. The consortia were awarded £25 million of government money, to which they added their own financial contributions.

In line with previous telehealthcare experiences, most of the Dallas activities take place at primary care and community level. It is believed that the motivation for hospitals to cooperate with other tiers of care and to keep people in the community lies in the lack of reimbursement for rehospitalisation within 30 days.

Furthermore, a recent reform in England introduced clinical commissioning groups led by primary care practices. Although the Quality Outcome Framework incentive system somewhat promotes patient self-monitoring and self-management, general practitioners have seldom seemed interested in telehealthcare. Ideally, National Health Service contracts would encourage groups of practices to take on a collective responsibility for both health and social care by providing additional services across the clinical commissioning groups using a range of local contract models, and ensuring coordination across these services. In this scenario, higher take-up of telehealthcare could be expected.

Overall a trend towards incentive reforms and reorganisation of care, promoting integrated care, was identified. It was also found that countries relying on fee-for-service payments (i.e. Estonia and Germany) had lower telehealthcare deployment. While fee-for-service are meant to increase general practitioners’ productivity, they do not provide the appropriate incentive environment for physicians to engage in complex cases or in the coordination of care. Neither does fee-for-service seem to incentivise information and communication technologies adoption.

Fee-for-service payments would seem to hamper the delivery of integrated care. An exception, however, is Denmark. Here, regional health and social care agreements represent command and control mechanisms which promote cooperation across tiers of care.

In contrast, capitation payments encourage general practitioners to provide the best possible preventive and long-term care, because this can be expected to reduce future costs. Information and communication technologies can help to achieve and manage these conditions.

A combination of capitation with outcome-based payments offers the benefits of both a payment-by-results and a pay-for-reporting incentive scheme. Paying by results is meant to keep patients in the community, hence its attractiveness. In addition, by including pay-for-reporting incentives, the role of information and communication technologies becomes prominent. This payment system is used in the UK and in some regions in Spain. Recent reforms in France and the Netherlands are taking payment in the same direction.

Looking at the clinical commissioning groups in England and comparing them to the care groups and the recently introduced bundled payment in the Netherlands, it seems that these two countries are taking a similar approach. They differ, however, in terms of telehealthcare deployment, given that these services are not currently being mainstreamed in the Netherlands. It may be argued that the Dutch bundled payment needs to have operated for longer for new services, such as telehealthcare, to become part of it. Instead, primary care in England has consolidated its role as the care coordinator for almost a decade. This acts as an enhancer for widespread telehealthcare deployment. In addition, the recent introduction of clinical commissioning groups as contractors could further strengthen this role. Indeed, one of the suggestions arising from the Nexes project in Catalonia pointed to this kind of payment as a way of promoting integrated care.

Diagnostic-related group payments for hospital care have been found across all countries. In some cases, they are limited to inpatient care, and in others they are also used for outpatient services, including telehealthcare services. Diagnostic-related group payments promote efficiency and early discharge, which at times may result in re-hospitalisation. However, diagnostic-related group payments do not necessarily promote coordination across tiers of care. An exception to this might be the established penalty under the payment-by-results scheme in England by which any patient rehospitalisation within thirty days of discharge is not reimbursed. Nonetheless, although such a penalty may act as an incentive at hospital level, it is not clear whether this incentive reaches the hospital staff who are making decisions about patient pathways.

An interesting case is that of Andalusia (Spain). Here, recent policies have aligned outcomes-based incentives to professionals in primary and hospital care. In addition, coordination teams involving practitioners from these two tiers of care have been established to support this process. This design is expected to promote the delivery of integrated care. At the moment, telehealthcare services are limited in Andalusia partly because of lack of funding. However, if this barrier was to be removed, this incentive mechanism would make Andalusia an interesting setting for telehealthcare deployment.

## Conclusion

The experiences researched have shown that trends towards better coordination and integrated care run alongside payment reforms designed to achieve these goals and telehealthcare developments, amongst others. Examples have been found in Denmark, the UK, and some regions in Spain, Italy and France.

Nonetheless, some of these incentive reforms should be regarded with caution. For instance, reimbursing telehealthcare services as an outpatient diagnostic-related group service may promote deployment but it may not promote cooperation across tiers of care in the long run. This form of payment may be useful during a transition period in which adoption needs to be promoted, but it may have to be replaced at some stage by other forms of payments which promote integrated care supported by telehealthcare.

The dominance gained by outcome-based incentives and bundled payments has also been identified (i.e. in England and the Netherlands, and it has been suggested as the way forward in Catalonia). If used from the outset, these incentives may promote integrated care, though their effectiveness remains to be seen.

Factors other than financial mechanisms should also be considered. For instance, if cooperation was the result of an initiative, health care professionals expressed high satisfaction even though it was not associated with financial retribution.

Power issues should also be considered, given that integrated care delivery embeds a service reorganisation. For instance, bundled payments shift the power base from hospitals to primary care practices, who act as contractors and coordinators. Penalising hospitals for re-hospitalisations within 30 days in order to promote this shift in the balance of power could have negative effects. In this regard, it is relevant to look not only at incentives at the organisational level but also at the individual level. For instance, aligning the incentives paid to professionals across tiers of care, as happens to some extent in Andalusia, could also result in greater cooperation and produce the desired outcomes.

Additional factors identified were those related to eHealth deployment. For instance, eGovernment mechanisms promoting the use of information and communication technologies in Denmark have also acted as drivers for eHealth deployment and interoperability, which would allow information sharing across tiers of care.

Another example was found in the Dallas programme in the UK, in which progress towards interoperability was a condition for funding.

Although information and communication technologies is not an end in itself, widespread information and communication technologies deployment is required to support the delivery of successful integrated care. Lack of interoperability therefore represents a major barrier which some countries are struggling to overcome. Interestingly, Estonia has placed less emphasis on integrated care. Here, once integrated care receives the final push required, the process will be facilitated by the interoperable information and communication technologies tools already in place supporting this process.

Denmark had a highly effective integrated governance approach to eHealth. Similarly, the eCare experience in Italy was highly multidisciplinary in terms of implementation and governance. These characteristics were identified as drivers, unlike the command and control aspects of other approaches.

The role of frameworks and funding to promote innovation were also relevant drivers. France, for instance, used a formula to promote the involvement of the private sector and enhance business models, based on public–private partnerships. In many places, however, it was found that initiatives originated at hospital level as traditionally this is where innovation is promoted, given the entrepreneurial environment and funding availability in these organisations. In this context, the challenge is how to transfer innovation to primary care level, which should take the lead in coordinating integrated care and keeping patients in the community. The savvy UK approach has been to provide funding to primary and community care, in order to diffuse innovation at this level.

Evidence about the impact of these interventions also played a relevant role, although in some settings more than others. For instance, though evidence was often limited, Scotland went ahead with wider telehealthcare deployment. England, instead required the evidence from the Whole Systems Demonstrators before deciding to mainstream.

In addition, in some cases (i.e. Catalonia or Denmark), the involvement of Health Technology Assessment agencies assisted the evaluation and dissemination of results. Thus, these agencies played a relevant role in promoting integrated care and telehealthcare adoption by introducing rigour into the evaluation methods and by consolidating evidence on the topic.

Finally, although a variety of factors which influence the development of integrated care and gaps in current incentive systems have been identified, policy-makers need to be aware that what may be possible in one care system may be impossible, at least in the short term, in another similar system, if the two differ in critical aspects.

## Reviewers

**Niels Boye**, Physician, Specialist in Internal Medicine, Endocrinology and Health Informatics, Klinisk Informatik, Aarhus, Denmark.

**George Crooks**, OBE, Medical Director NHS 24, Glasgow, UK.

One anonymous reviewer.

## Figures and Tables

**Table 1.**
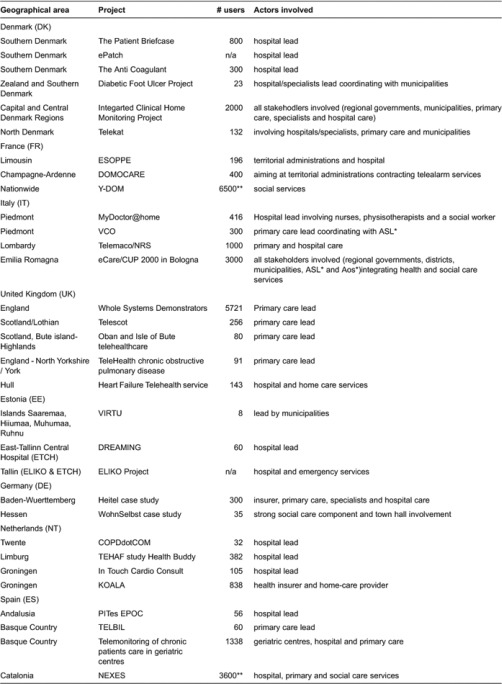
Telehealthcare deployment across the countries studied

Source: author's analyses of the eight country studies produced [30–37].

*ASL are local health enterprises; AOs are Public Hospital Enterprise.

**Numbers in italics represent target number of users as opposite to actual users.

**Table 2.**
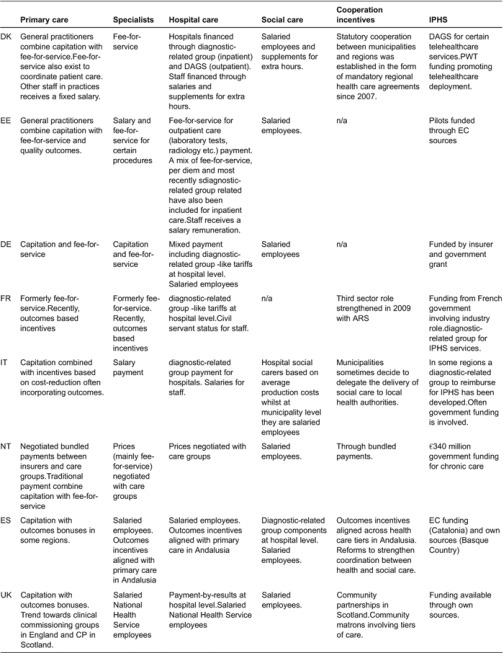
Organisation and incentive schemes in eight countries

Source: Author's analyses of the eight countries' studies produced [30–37].
